# Supplementary material for: Hypoxia-Inducible Factor-1α, a Novel Molecular Target for a 2-Aminopyrrole Derivative: Biological and Molecular Modeling Study
Source: Cancers (Basel). 2025 Dec 30;18(1):115. doi: 10.3390/cancers18010115 (PMC12784696; doi:10.3390/cancers18010115)

Figure 2. Pro-apoptotic activity of 2-ANPC in epithelial cancer cell lines.

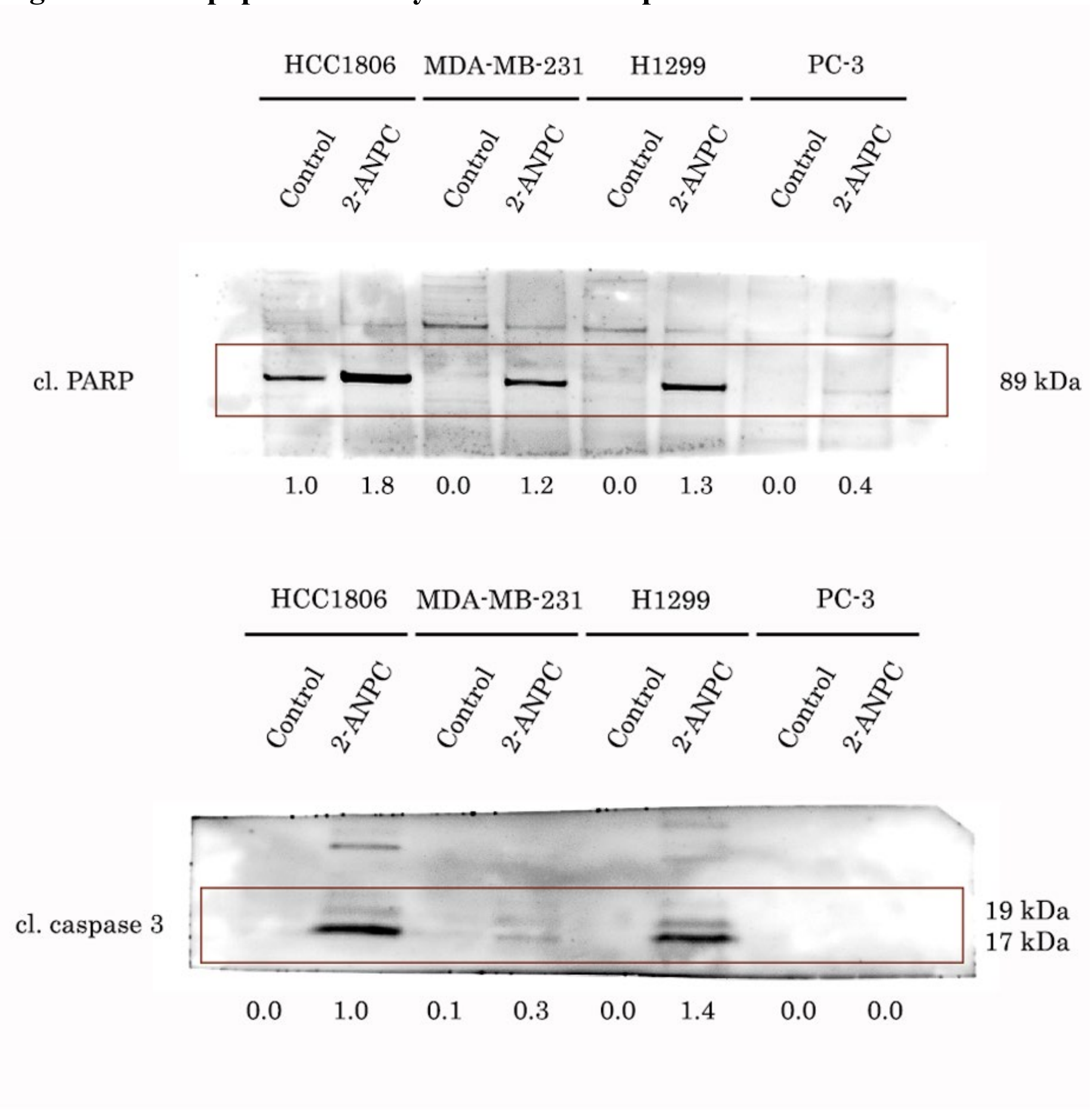

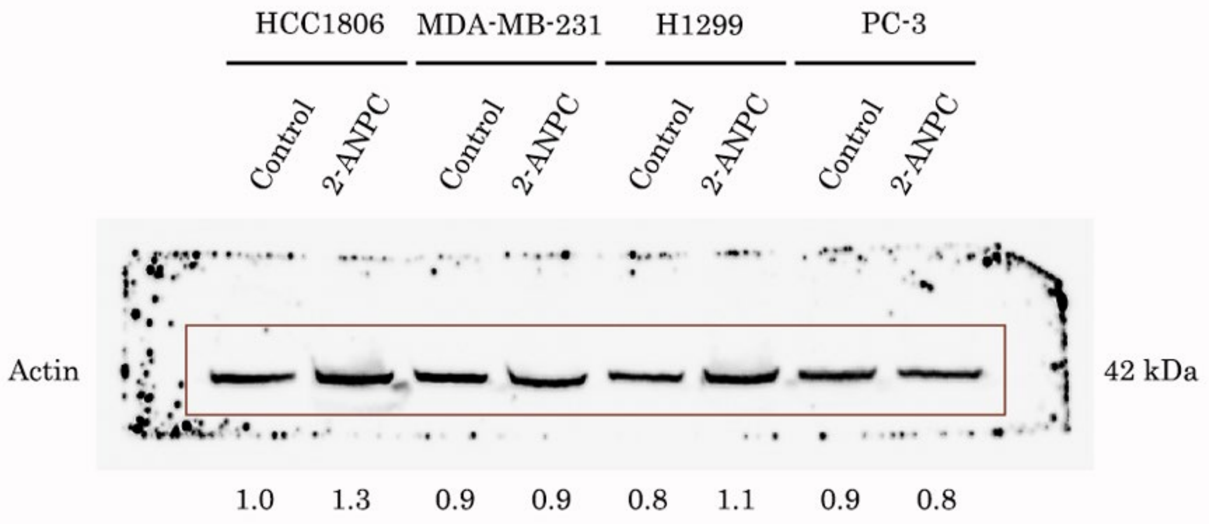

HCC1806
MDA-MB-231
H1299
PC-3
Control
2-ANPC
Control
2-ANPC
Control
2-ANPC
Control
2-ANPC
Actin
42 kDa
1.0
1.3
0.9
0.9
0.8
1.1
0.9
0.8

Figure 3. 2-ANPC decreases expression of HIF-1α in epithelial cancer cell lines by reducing its stability and promoting the proteasome-dependent degradation.

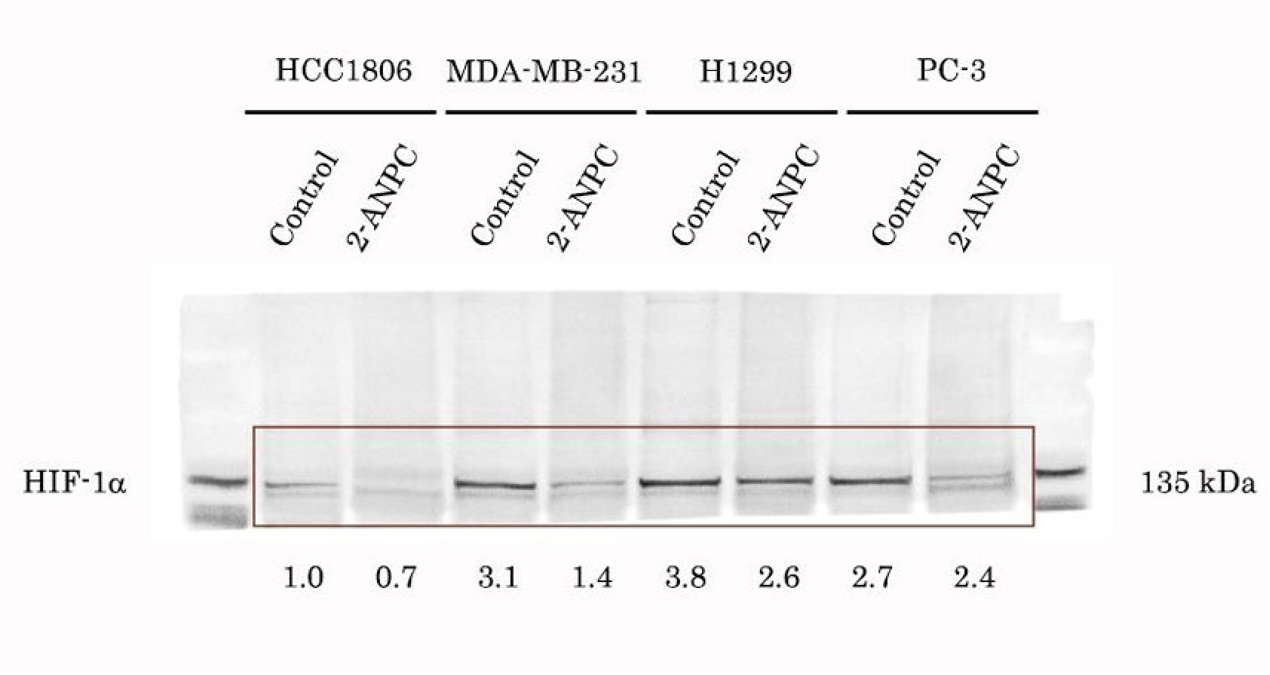

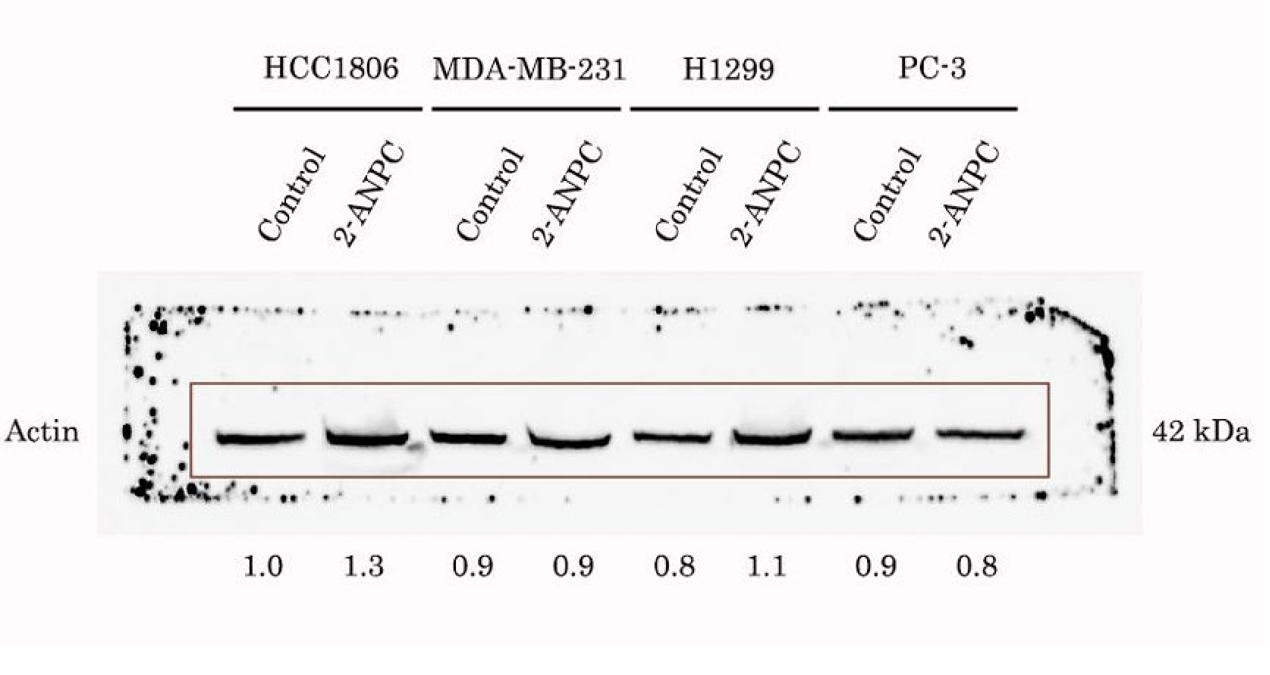

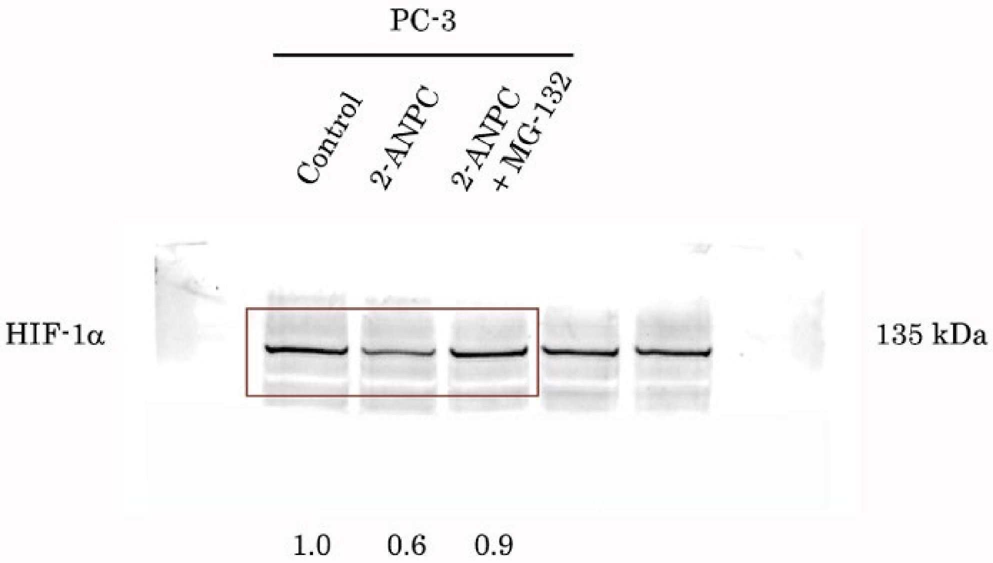
PC-3
Control
2-ANPC
2-ANPC + MG-132
HIF-1α
135 kDa
1.0
0.6
0.9

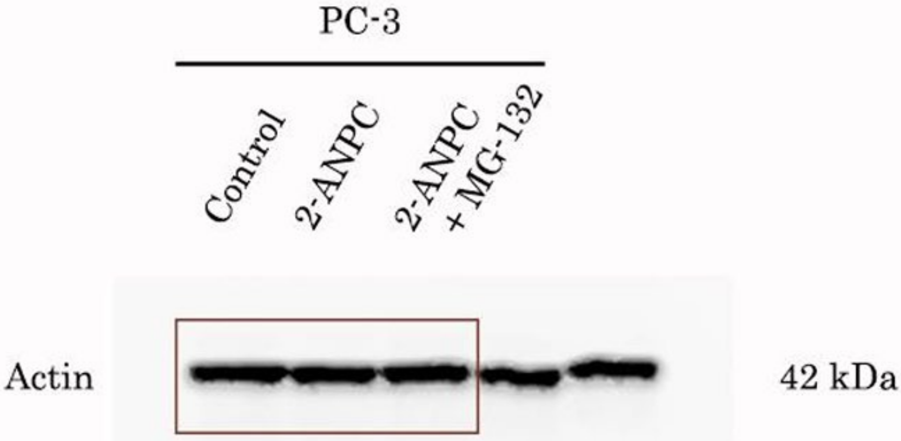
PC-3
Control
2-ANPC
2-ANPC + MG-132
Actin
42 kDa
1.0
0.9
1.0

Figure 4. 2-ANPC decreases HIF-1α's stability under hypoxic conditions.

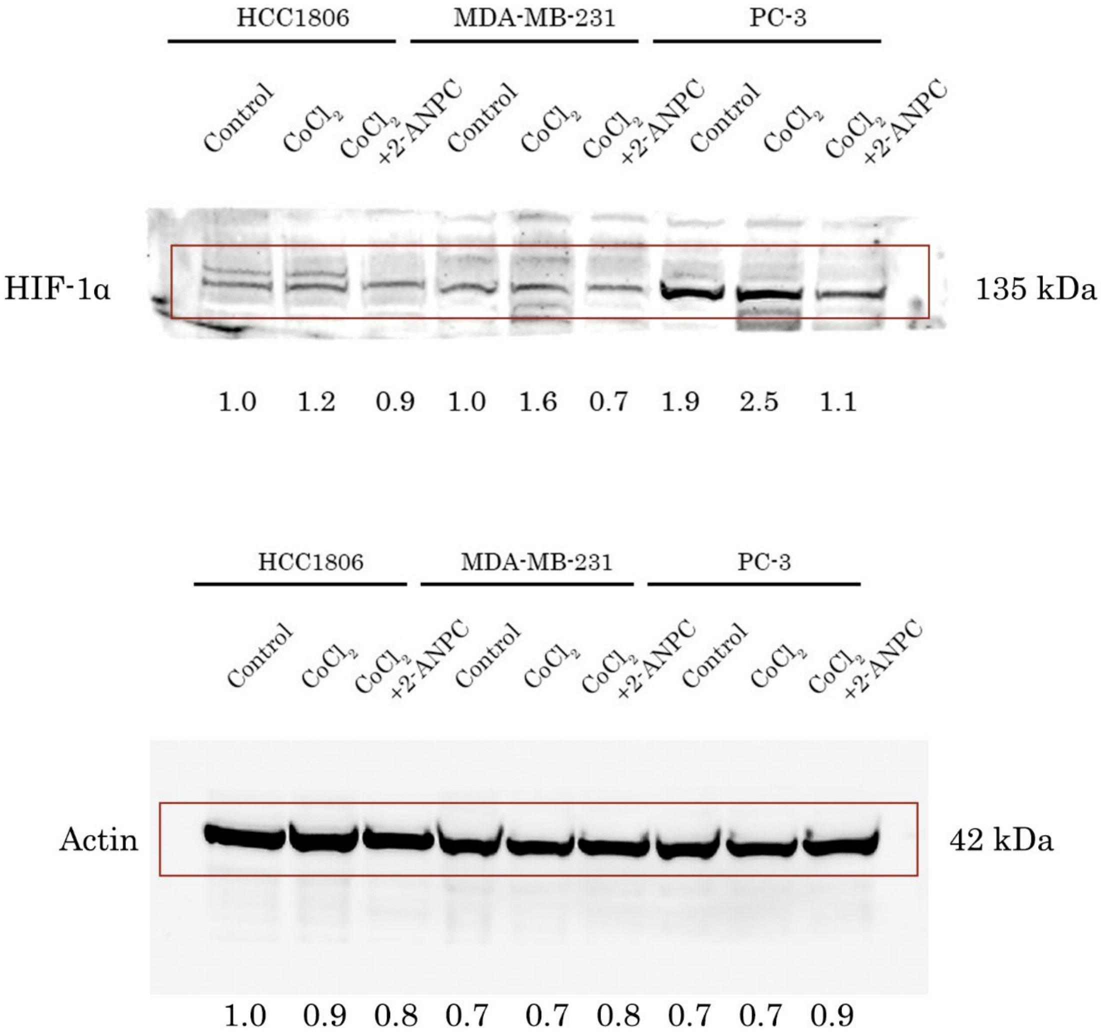

Figure 7. 2-ANPC decreases HIF-1α expression in 4T1 breast cancer cells in vivo and in vitro.

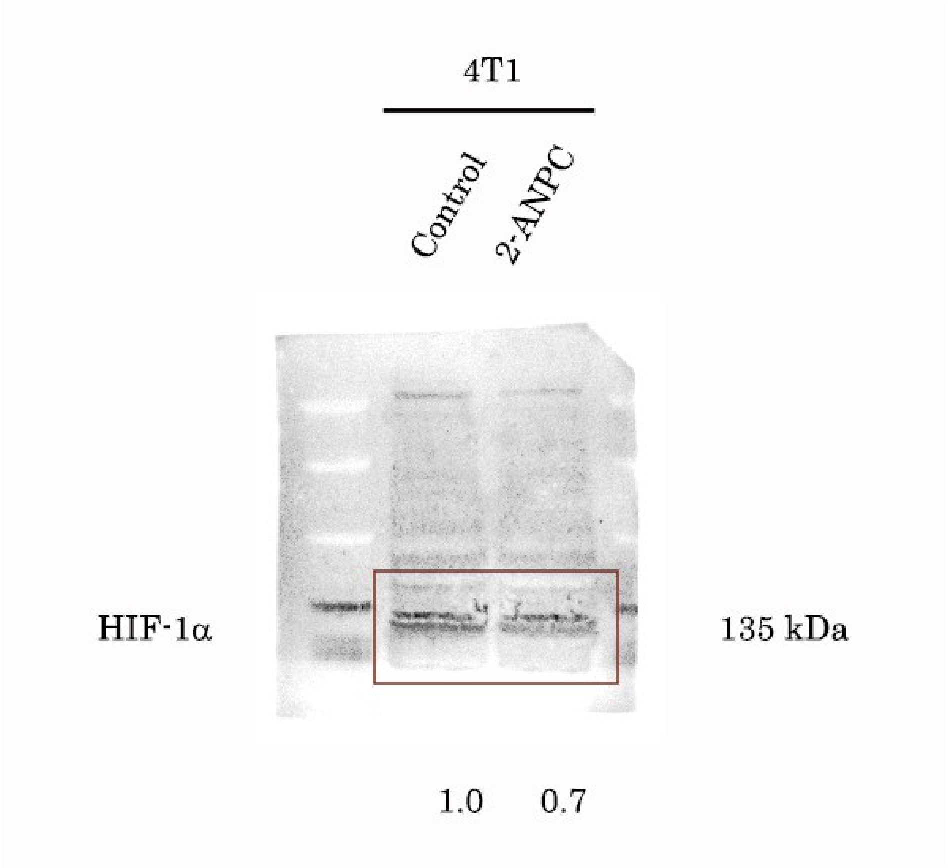

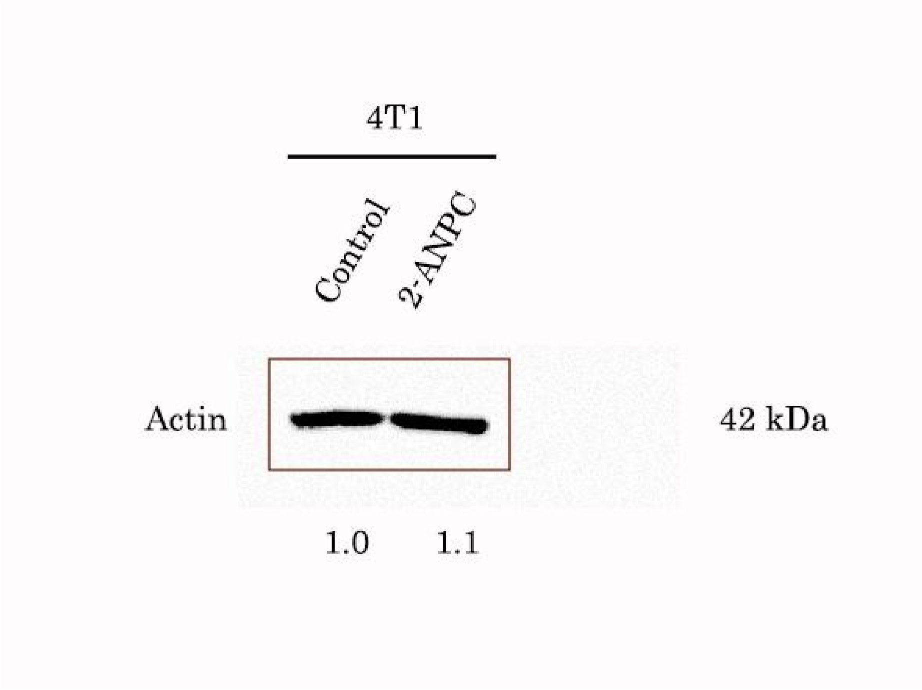

Supplementary Figure S1. Anti-proliferative and pro-apoptotic activity of 2-ANPC in 4T1 cell line.

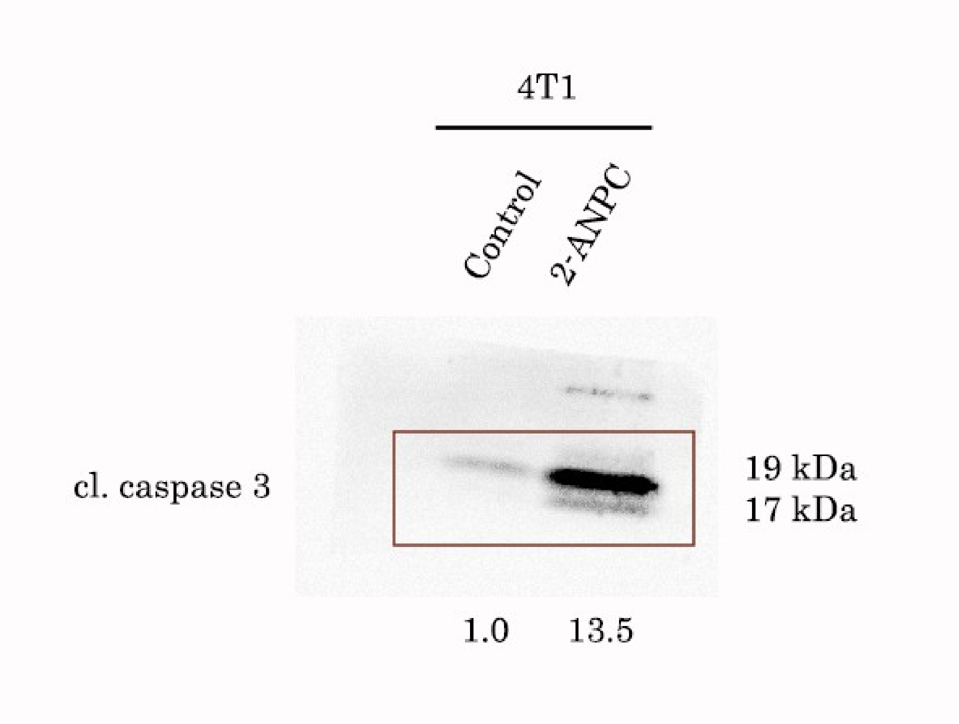

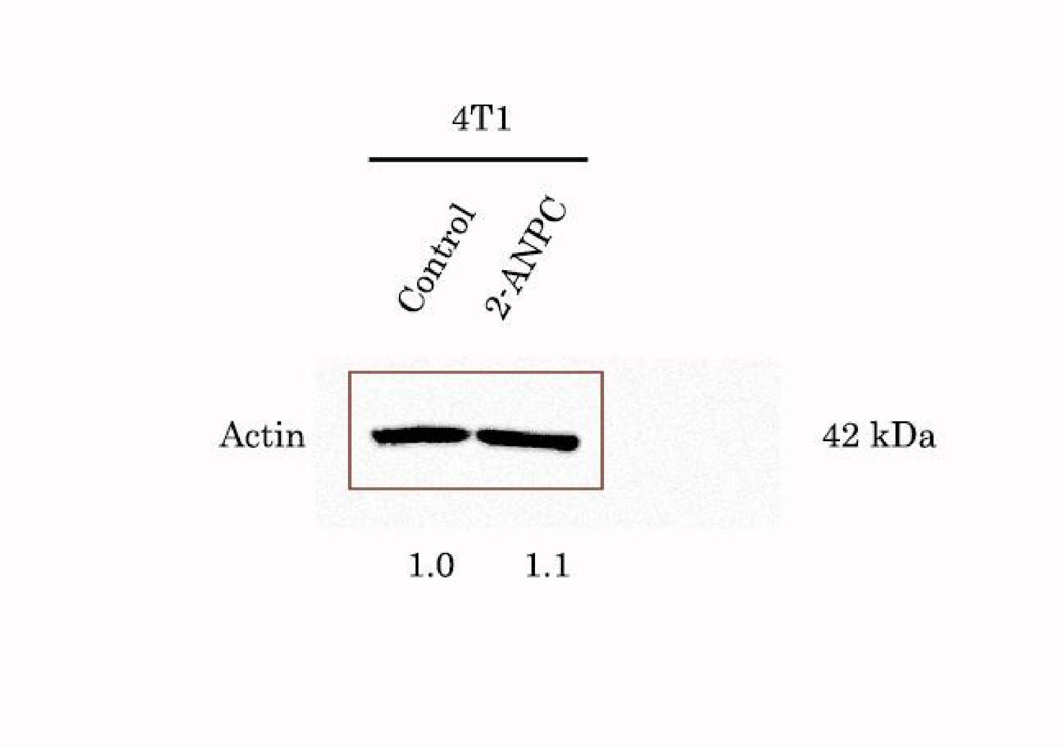

Supplement: Supplementary file 1 [file cancers-18-00115-s001.zip › cancers-4051266-File S1.pdf]
