# Supplementary material for: Hypoxia-Inducible Factor-1α, a Novel Molecular Target for a 2-Aminopyrrole Derivative: Biological and Molecular Modeling Study
Source: Cancers (Basel). 2025 Dec 30;18(1):115. doi: 10.3390/cancers18010115 (PMC12784696; doi:10.3390/cancers18010115)
Supplement: Supplementary file 1 [file cancers-18-00115-s001.zip › cancers-4051266-supplementary-revised.pdf]

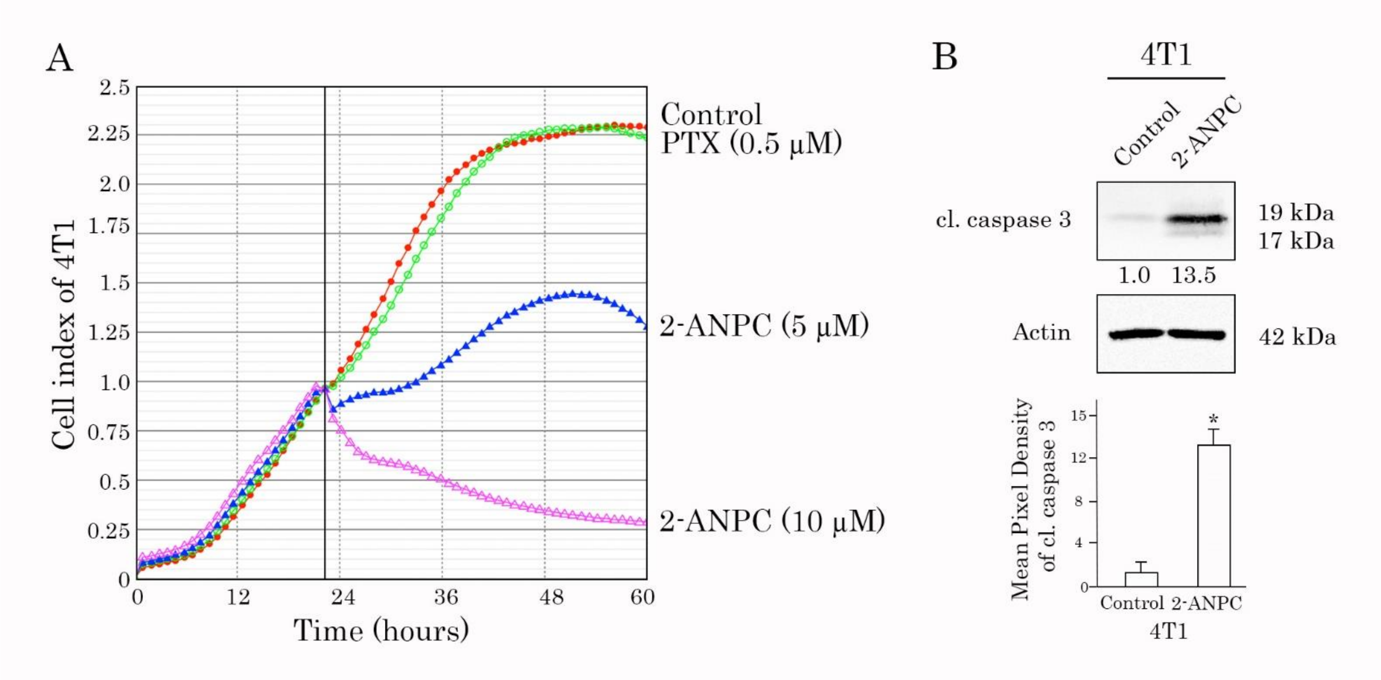

Supplementary Figure S1. Anti-proliferative and pro-apoptotic activity of 2-ANPC in 4T1 cell line. (A) Changes in growth kinetics of 4T1 cells and treatment with DMSO (control) and 2-ANPC. 4T1 cells (0.5×10^5 /ml) were seeded into the wells of an E-Plate L8 PET cassette and installed in the iCELLigence cell growth kinetics system (ACEA Biosciences, San Diego, CA, USA). Cells were allowed to attach and grow for the following 24 h. Afterwards, 2-ANPC at 10 µM was added to the cell culture. DMSO-treated cells served as the control. Cell proliferation index values were recorded every hour throughout the experiment. RTCA Software version 1.0 (ACEA Biosciences, Inc., San Diego, CA, USA) was used to analyze the data. (B) 2-ANPC increases the expression of the cleaved form of caspase-3 in 4T1 breast cancer cells in vitro. Cells were treated with 2-ANPC (10 µM) for 48 h and subjected to western blotting analysis to examine the expression of the cleaved form of caspase-3. Actin staining was used to show the comparable amounts of protein loaded into each sample. The figure below shows the quantification of caspase-3 cleavage in cells, based on mean pixel density. Values are means ± SD, N = 3. Asterisk indicates significant difference compared to relevant controls ($p < 0.0001$; one-way ANOVA).

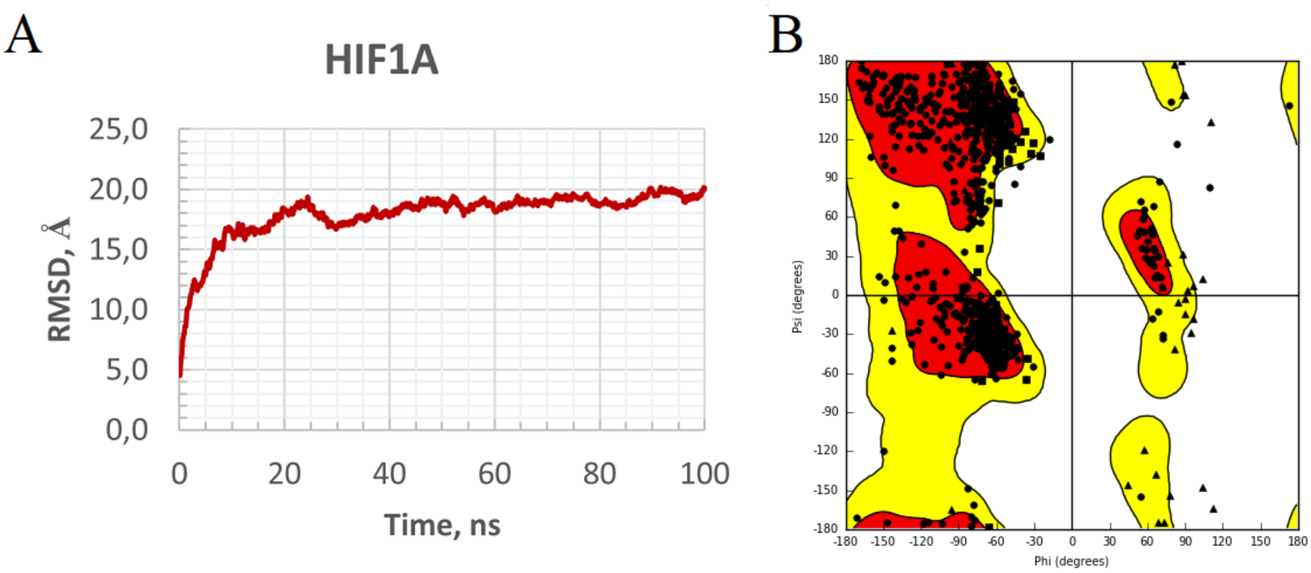

Supplementary Figure S2. (A) Root mean square deviation of atomic positions plot for the hypoxia-inducible factor (HIF)-1α after its modelling. (B) Ramachandran plot for the chosen snapshot at 99.6 ns.

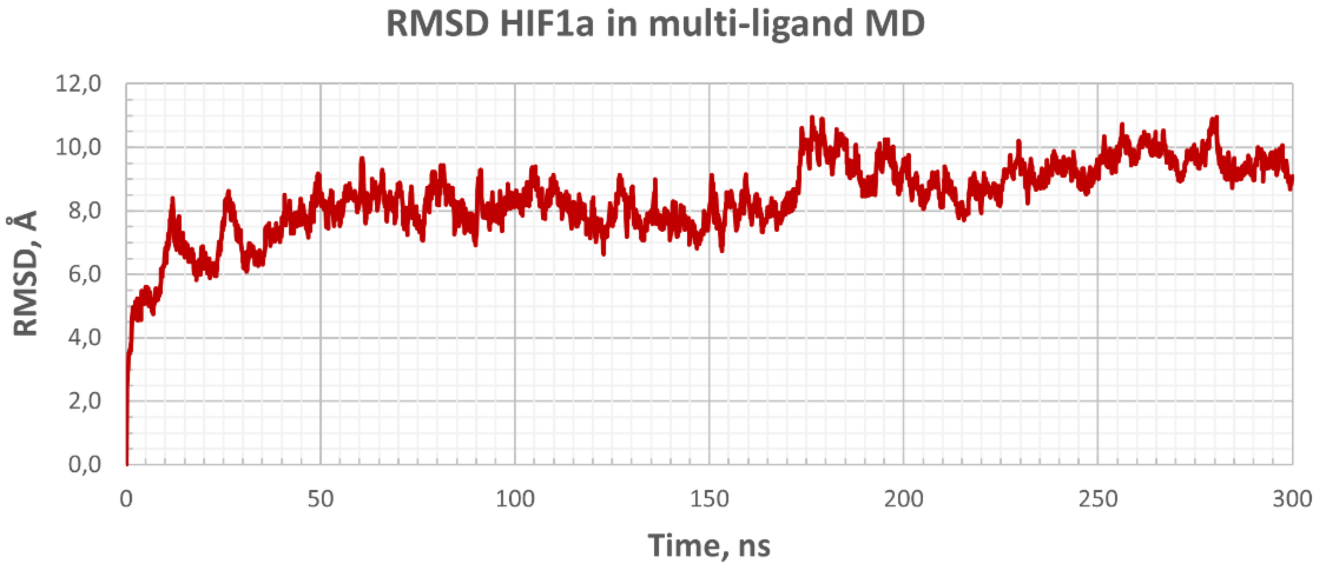

Supplementary Figure S3. Root mean square deviation of atomic positions plot for the hypoxia-inducible factor (HIF)-1α for the multi-ligand MD simulations.

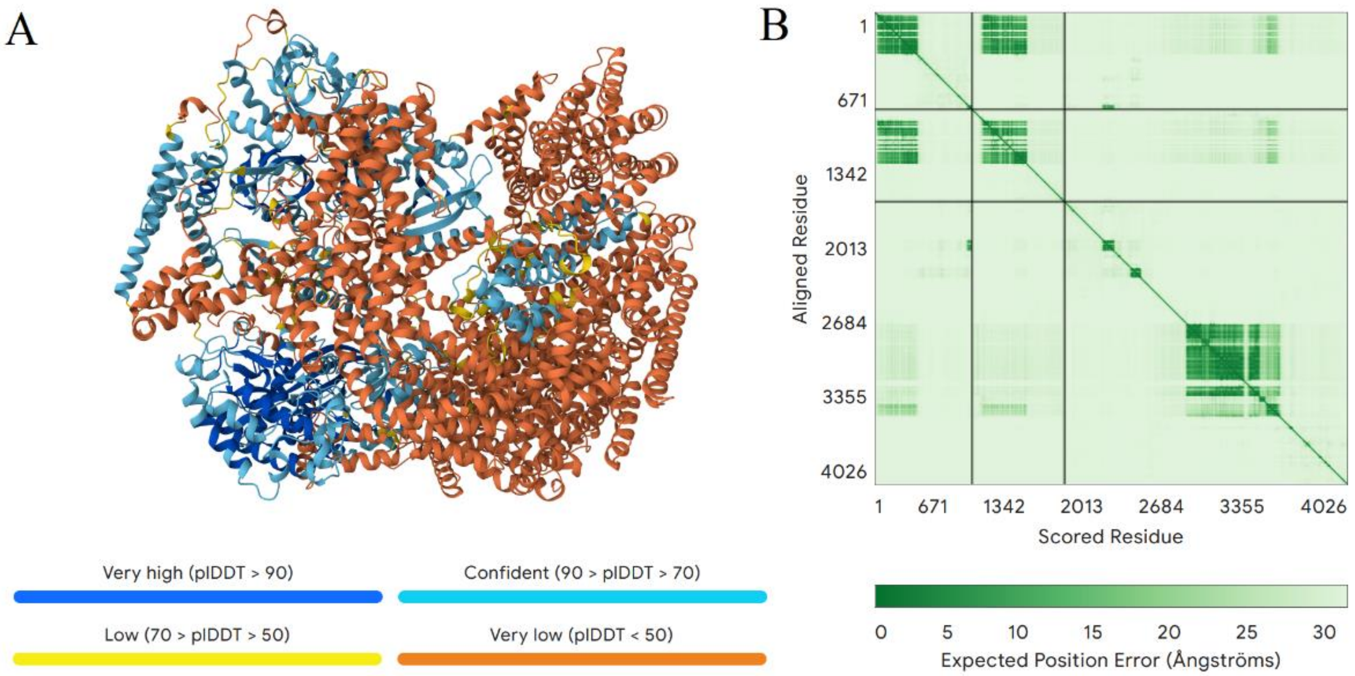

Supplementary Figure S4. (A) pLDDT confidence scores for predicted HIF-1 protein complex. (B) Expected Position Error for the predicted HIF-1 protein complex.

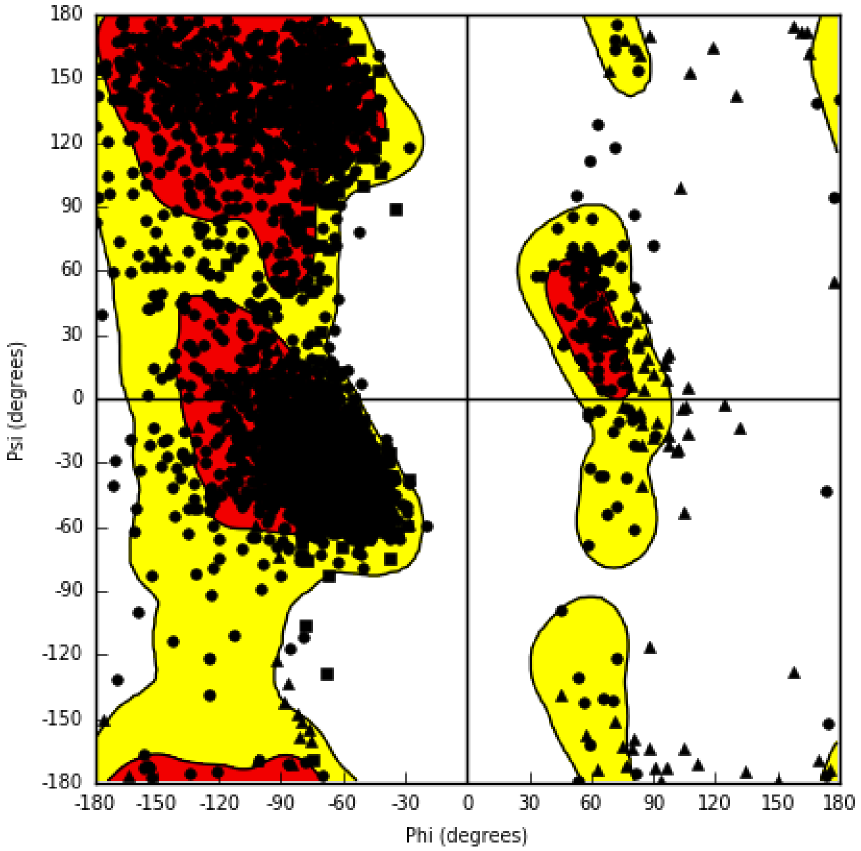

Supplementary Figure S5. Ramachandran plot for the chosen median of the MD clusters.

Supplementary Table S1. Primers for quantitative polymerase chain reaction

Gene	Forward	Reverse
HIF1A	5´-TGCAACATGGAAGGTATTGC-3´	5´-TTCACAAATCAGCACCAAGC-3´
GAPDH	5´-GACCACAGTCCATGCCATCA-3´	5´-TCCACCACCCTGTTGCTGTA-3´

Supplementary Table S2. Docking results for 2-ANPC in HIF-1α

Binding site	SiteScore	Docking score, kcal/mol	Glide Emodel, kcal/mol	IFD score, kcal/mol	Pi-interactions	H-bond interactions	Hydrophobic interactions
1	1.060	-9.788	-103.890	-1737.20	PHE168, HIS286	GLU225, THR302, GLN304	PHE168, THR290, MET450, PRO452
2	1.032	-8.180	-91.853	-1734.50		Lys759	ILE639, GLU704
3	1.028	-7.358	-78.309	-1733.51		ASP417	LYS251, GLU277, LEU354, PRO468, LEU470
4	1.102	-9.785	-99.307	-1736.10	HIS229, PHE540	ARG170, THR188, TRP189, TYR325	LYS190, PRO230, THR322, ILE324, VAL336, PHE540
5	NA	-6.073	-69.496	-1076.68		THR260, MET263, HIS348, LYS370	THR260, GLU261

Supplementary Table S3. Docking results for 2-ANPC in HIF-1

Binding site	SiteScore	Docking score, kcal/mol	Glide Emodel, kcal/mol	IFD score, kcal/mol	Pi-interactions	H-bond interactions	Hydrophobic interactions
1	1.003	-9.420	-92.648	-503.11	HIF-1β: HIS138	HIF-1α: LYS71, ASP74, ASN206	HIF-1α: PRO117 LEU142 THR188
2	1.005	-7.136	-88.646	-805.20	HIF-1α: LYS12 (pi-cation)	p300: LYS1006 ARG1015 GLU1026	HIF-1α: MET1, LYS12<br>p300: ILE1023
3	1.032	-10.084	-93.223	-865.62	HIF-1β: PHE74	p300: LYS1291 SER1295 THR1322	HIF-1β: PHE74
4	0.987	-6.114	-78.887	-522.10	HIF-1β: LYS58<br>(pi-cation)	p300: ASP1384 ASP1614	HIF-1β: LYS58, PHE59<br>p300: VAL1325
5	0.906	-7.398	-73.855	-578.52		HIF-1β: ASP50, ASP52<br>p300: ARG1410	HIF-1β: LEU47, PHE49
6	1.183	-10.731	-123.049	-1065.40	p300: HIS1451 (pi-pi and pi-cation)	p300: GLN1455, THR1446	p300: ILE1457, PRO1458, LEU1463
